# Percutaneous iliosacral screw fixation in unstable pelvic fractures

**DOI:** 10.11604/pamj.2017.27.244.11506

**Published:** 2017-08-03

**Authors:** Hicham Bousbaa, Mohammed Ouahidi, Jamal Louaste, Mourad Bennani, Tawfiq Cherrad, Hassan Jezzari, El Houssine Kasmaoui, Khalid Rachid, Laarbi Amhajji

**Affiliations:** 1 Department of Orthopaedics & Traumatology, Military Hospital Moulay Ismail, BP 50000 Meknes, Morocco

**Keywords:** Unstable pelvic fracture, sacroiliac joint, sacral fracture, percutaneous iliosacral screws, pelvic fixation

## Abstract

Surgical treatment of unstable pelvic fractures Type C, has a vertical instability that is not controlled by traction and supine; therefore, orthopedic and functional treatments undertaken by default are sources of complications. The closed reduction with percutaneous sacroiliac fixation solves the problem of vertical instability; but at the cost of learning the method. Five patients with unstable pelvic fractures; were treated by percutaneous sacroiliac fixation. This reliable and useful method in the stabilization of unstable pelvic fractures. Good functional results are predictable based on the severity of pelvic fractures and associated injuries. The low rates of complications and the minimally invasive nature are the advantages of this method.

## Introduction

Surgical treatment of unstable pelvic fractures Type C has a vertical instability; that is not controlled by traction and supine [1]; therefore, orthopedic and functional treatments undertaken by default are sources of complications [2]. The closed reduction with percutaneous sacroiliac screw fixation solves the problem of vertical instability; but at the cost of learning the method. [3]. The objective of this study is to evaluate the results of five patients with these fractures, treated by sacroiliac screws.

## Methods

**Figure 1:**
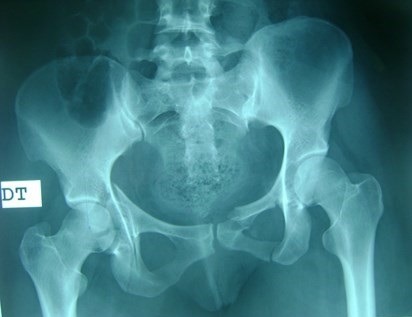
The first case with a fracture of the right anterior column associated with a fractured left the obturator ring and left sacroiliac disjunction

**Figure 2:**
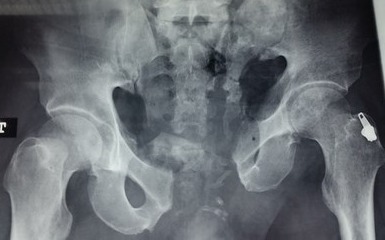
The second case with a pubic disjunction associated with a fracture of the left sacred aileron

**Figure 3:**
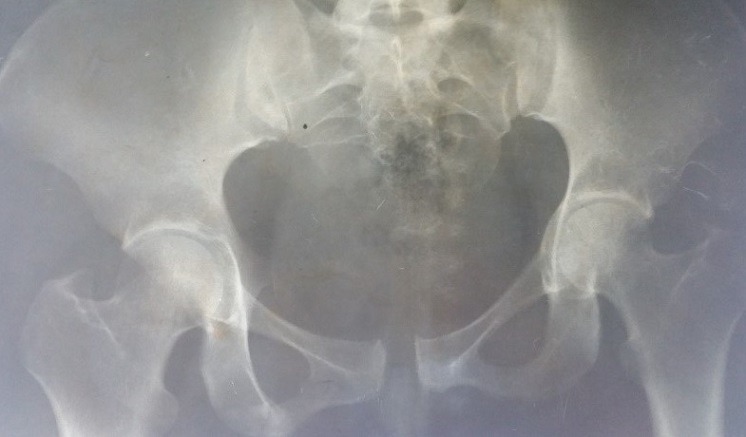
The third case with a pubic disjunction and disjunction sacroiliac

**Figure 4:**
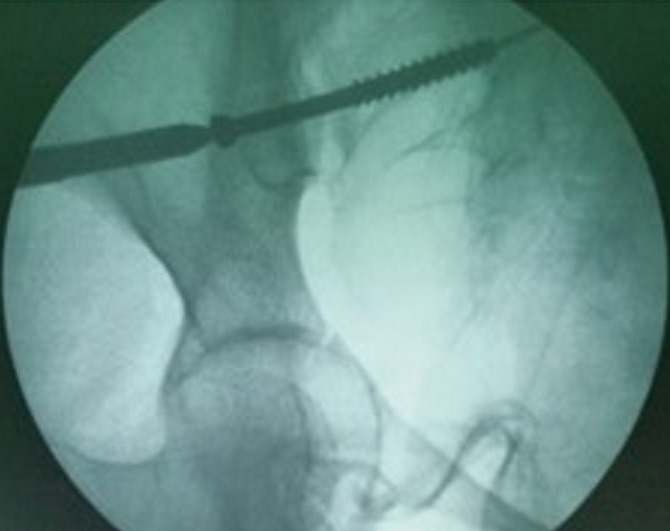
Sacroiliac screw under fluoroscopic control

**Figure 5:**
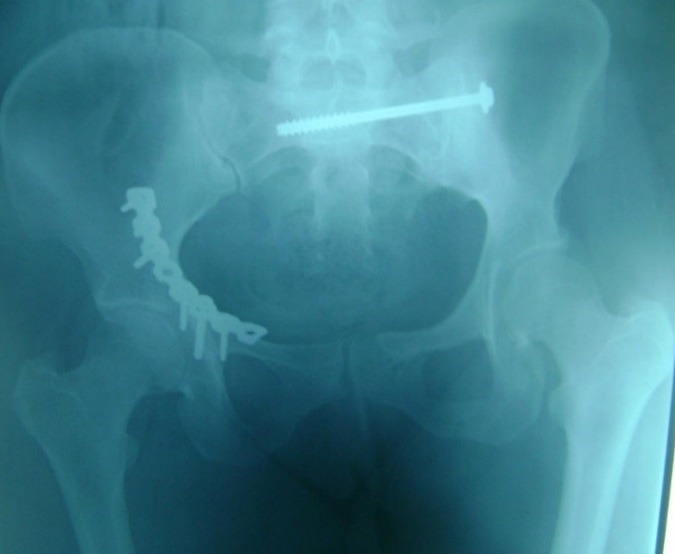
Postoperative control of the first patient who received a plate fixation of right acetabular fractures and then percutaneous screwing of the left sacroiliac disjunction

**Figure 6:**
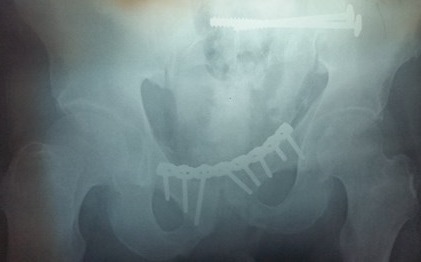
Postoperative control of the second patient who received an osteosynthesis of pubic disjunction and sacroiliac screw of the left sacral wing fracture

**Figure 7:**
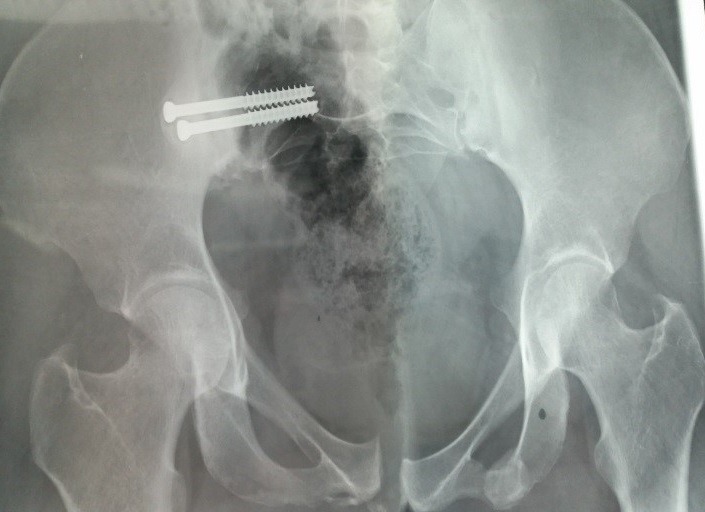
Postoperative control of the third case which received a double sacroiliac screw

Between 2012 and 2014, 05 patients with unstable pelvic fractures; were treated by percutaneous sacroiliac fixation. This is 2 women and 3 men; the average age was 38 ± 10 years, all the patients were treated by percutaneous screwing. The initial radiological evaluation consisted of a radiograph of the pelvis and a CT of the pelvis; which allowed the classification of these fractures according to AO Tile modified classification; and all five patients had a fractured pelvis type C. The first patient had a fracture of the anterior column with a fracture of the obturator ring; and left sacroiliac disjunction (Figure 1). The second patient had a pubic disjunction associated with a fracture of the sacral aileron (Figure 2). The third case was suffering from pubic disjunction associated with sacroiliac disjunction (Figure 3). The last two cases had an obturator ring fracture associated with sacroiliac disjunction (Figure 4). In all patients there was a high-energy trauma, and all of these patients had multiple lesions associated; namely a head injury in two patients; an ankle fracture in two patients, a leg fracture in a patient. By cons they had no urological injury or a vascular - nervous. All patients have had surgery by the same surgeon under spinal anesthesia on a fracture table, the pool end of the table; with a pull of both lower limbs as well as calluses fessiers.il must absolutely ensure that nothing interferes with the image intensifier in the implications and Outlet Inlet. We started by reducing the anterior displacement in all patients. The operating time was about 48 hours in all patients except in one case with neglected fracture 10 months. So since the sacrum is oblique at 45°, it is seen from the front on the Outlet incidence; we will guide the drill to avoid the sacred holes; then, the sacrum is seen from above on the Inlet incidence there by guide the drill to the vertebral body and prevent the spinal canal. Two sacro iliac screws 7 mm in diameter in four patients were performed (Figure 5, Figure 6, Figure 7). All patients were put on anticoagulant to resume walking. The rehabilitation was started after surgery with isometric contractions; then passive and active gradually. Putting e Full support is done after the 12^th^ week.

## Results

The average hospital stay depends on associated lesions; it averaged 15 days. Mean follow-up was 12 months (8 months -30 months), medium term full loading 9 ± 3 weeks. No secondarily revision surgery was necessary; an additional anterior stabilization was necessary in two patients. We have not encountered infection or nerve damage, or cases of nonunion of the posterior arch; not those of the pubic rami. Apart from the patient with a sacred nonunion; consolidation was not certain. According to the criteria of Matta to reduce pelvic fractures all patients had a satisfactory reduction. They were able to resume all their activities; except the patient with the neglected fracture of the sacrum.

## Discussion

Unstable fractures of the pelvic ring injuries are serious, associated with a high rate of morbidity and mortality [1]. Early surgical stabilization showed a reduction in pain, early mobilization of the patient, and in the end ensures good functional recovery in the long term [2]. Conservative treatment does not offer precise reduction and confines the patient to bed with all possible complications [3]. The purpose of this study was to describe the technique of percutaneous sacroiliac screw in unstable pelvic fractures; using conventional fluoroscopy and evaluate the perioperative complications and radiological results. Routt [4] was the first to describe the technique of stabilizing the sacroiliac dislocation in the supine position under fluoroscopy. He demonstrated that it is a reliable and reproducible technique that can significantly reduce complications observed in the open techniques without sacrificing stability. Mears et al. [5] have shown in vitro that mechanically a single screw offers the same biomechanical stability than an intact basin. Yinger K and Van Zwienen CM [6, 7], in their two comparative studies indicate a high biomechanical stability with double sacroiliac screw in fractures with vertical instability. All patients were treated was using materials commonly available of osteosynthesis with a simple fluoroscopic guidance, probably the crucial element in the placement of screws and obtaining a good reduction of the fracture or joint sacroiliac and then confirming the ideal position of the sacroiliac screw by an X-ray profile intraoperative as she was described by Routt. The disadvantage of the percutaneous technique is that the Direct reduction is impossible. So an anatomic reduction is difficult. The functional outcome was always associated with poor reduction of the posterior arch of the pelvis (Table 1). Postoperative reduction was good to excellent most cases (80%). Similar results were previously published [8–13]. Previous studies report a secondary displacement rate of 3% to 5% and nerve damage from 0 to 8% [5, 8, 14–17]. This is consistent with our data, even if the distribution of the severity of fractures identity could not be due to the use of different classification systems. The large share of complications related to the patient, not the technology itself (Table 2). Although various techniques called scanno-guided or navigated insertion systems of computer screws have been described [18–21], the emergency use in the treatment of trauma patients is controversial. Especially with all their time to set up in the context of the emergency [18]. In some studies, the operating time can be reduced by the use of computer-based systems [21–23], others are reporting an increase in time of the procedure [19, 23]. Indeed, the preparation of the configuration for navigation takes extra time. When navigation is based on preoperative CT, potential displacement fracture between scanner and surgery are rare. Clearly, inland systems have the advantage of a fluoroscopic time reduction [18, 19, 21, 23] and will improve the operating time in the future. Although greater accuracy of screw placement was described for the computer-assisted surgery [19, 20, 23] others have found no difference between the system or sailed scanno-guided and conventional placement of screws; or on the occurrence of neurological injury [17, 18, 20].

**Table 1:** Comparison of radiological results

	Pohle-mann et al. [8]	Matta and Tornetta [9]	Tornetta and Matta [10]	Tornetta et al. [11]	Suzuki et al. [12]	S.A Khaled [13]	This series
**Excellent %**	63	67	75	76	51	71,4	80%
**Good %**	24	28		21	23	20,8	0
**Acceptable %**	13	4		3	16	7,8	20%
**Poor %**	0	1		0	10	0	0

**Table 2:** Comparison of complications

	Routt et al. [14]	Nork et al. [15]	Van Den Bosch et al. [16]	Moed and Geer [17]	S.A. Khaled [13]	This series
**Number of patients**	177	13	88	49	77	5
**Age (years)**	32	-	-	14-71	32,6(9-70)	38 10
**Followed (month)**	15(6-48)	-	-	-	37,4	12(8-30)
**Neurological or vascular injury**	0	0	7	0	0	0
**Infection or Hematoma**	0	0	0	0	0	0
**Secondary displacement**	0	0	1	2	2	0
**Pseudarthrosis**	0	0	0	0	0	0

## Conclusion

Reliable and useful method in the stabilization of unstable pelvic fractures. Good functional results are predictable based on the severity of pelvic fractures and associated injuries. The low rates of complications and the minimally invasive nature are the advantages of this method.

### What is known about this topic

- Surgical treatment of unstable pelvic fractures Type C has a vertical instability; that is not controlled by traction and supine [**1**]; therefore, orthopedic and functional treatments undertaken by default are sources of complications;
- The closed reduction with percutaneous sacroiliac screw fixation solves the problem of vertical instability;
- The cost of learning the method.

### What this study adds

- Reliable and useful method in the stabilization of unstable pelvic fractures;
- Good functional results are predictable based on the severity of pelvic fractures and associated injuries;
- The low rates of complications and the minimally invasive nature are the advantages of this method.

## Competing interests

The authors declare no competing interest.
